# Exploring the Therapeutic Potential of Antibiotics in Hyperglycemia-Induced Macrophage Dysfunctions

**DOI:** 10.3390/antibiotics14020198

**Published:** 2025-02-14

**Authors:** Montira Yossapol, Piyarat Srinontong, Worapol Aengwanich, Monchaya Panil, Supissara Somsup, Justice Opare Odoi, Jaroon Wandee

**Affiliations:** 1 Faculty of Veterinary Sciences, Mahasarakham University, Mahasarakham 44000, Thailand; montira.y@msu.ac.th (M.Y.); piyarat@msu.ac.th (P.S.); worapol.a@msu.ac.th (W.A.); 2 Bioveterinary Research Unit, Mahasarakham University, Mahasarakham 44000, Thailand; 3 Allpet Small Animal and Exotic Hospital, Bang Khae, Bangkok 10160, Thailand; mmonchaya.pan@gmail.com; 4 101 Small Animal Hospital, Muang Roi Et, Roi Et 45000, Thailand; somsup.supissara@gmail.com; 5 Animal Health Division, Animal Research Institute, Council of Scientific and Industrial Research, Accra P.O. Box AH20, Ghana; odoij19890606@gmail.com

**Keywords:** hyperglycemia, macrophages, antibiotics, phagocytosis, diabetes

## Abstract

**Background:** Diabetes mellitus exacerbates immune dysfunction, leading to higher susceptibility to infections. This study investigated the effects of antibiotics on macrophage functions under high glucose conditions to mimic a diabetic context. **Methods:** Using murine macrophage cell line RAW 264.7, the present study evaluated the cytotoxicity, phagocytosis, bactericidal activity, and pro-inflammatory cytokine production after treatment with four antibiotics: oxytetracycline, ciprofloxacin, sulfamethoxazole–trimethoprim, and cefotaxime. **Results:** All antibiotics demonstrated no cytotoxicity across 1×–8× MIC concentrations. Hyperglycemia significantly impaired macrophage phagocytosis and bactericidal activity while inducing pro-inflammatory mediator markers, *IL-1, IL-6, TNF-α,* and *iNOS*. Only ciprofloxacin significantly improved phagocytic achieving levels comparable to the low glucose control. Treatments with ciprofloxacin, sulfamethoxazole–trimethoprim, and cefotaxime significantly enhanced bactericidal activity without altering the pro-inflammatory cytokine profile. **Conclusions:** These findings underscore the negative effect of high glucose on macrophage functions and suggest that ciprofloxacin may be a potential therapeutic option for diabetes-associated infections.

## 1. Introduction

Diabetes mellitus (DM) is a chronic metabolic disorder characterized by persistent hyperglycemia resulting from defects in insulin secretion, insulin action, or both. The prevalence of diabetes has been rising globally, with an estimated 828 million adults affected in 2022 [1], while projections indicate a 25% increase by 2030 and a 51% increase by 2045, posing significant public health challenges [2]. Chronic hyperglycemia in diabetes is associated with the development of long-term complications, including cardiovascular diseases, nephropathy, retinopathy, neuropathy, ulceration, and secondary bacterial infection, all of which increase the risk of death in diabetic patients [3]. In particular, diabetes with bacterial infections significantly increases hospitalization rates and contributes to higher morbidity and mortality among diabetic patients. Diabetes was found to account for approximately 6% of infection-related hospitalizations and 12% of infection-related deaths [4,5,6]. This has turned into a medical problem that requires immediate attention. The bacteria most commonly associated with chronic complications include *Escherichia coli*, *Enterobacter* spp., *Staphylococcus aureus*, *Klebsiella* spp., *Acinetobacter* spp., and *Enterococcus* spp. [4]. Increased susceptibility to infections in diabetic patients has been linked to hyperglycemia-induced impairments in innate and adaptive immune responses [7].

Dysfunction of phagocytic cells has been observed in diabetes, resulting in reduced bacterial clearance, higher intracellular bacterial burden, and an overall heightened susceptibility to infections [8]. Macrophages, key phagocytes in the inflammatory response, are classified into two types: pro-inflammatory (M1) and anti-inflammatory (M2). M1 macrophages dominate the early phase of inflammation by engulfing pathogens and releasing pro-inflammatory mediators like IL-1, IL-6, TNF-α, IFN-γ, and iNOS, which help eliminate the threat. In the later stages, M2 macrophages release anti-inflammatory mediators such as IL-4, IL-10, and TGF-β to initiate tissue healing and prevent further damage from prolonged inflammation [9]. However, in the context of diabetes, particularly under high glucose levels, the function of macrophages is significantly altered. Hyperglycemia induces a pro-inflammatory phenotype in macrophages, which is characterized by enhanced pro-inflammatory mediator expressions, including IL-1, IL-6, TNF-α [10,11,12], and NO [13]. This further sustains M1 macrophage polarization, ultimately leading to chronic inflammation [14]. Furthermore, previous studies showed that hyperglycemia impaired both the phagocytic function [11,15] and bactericidal activity [10,11] in macrophages. Thus, enhancing macrophage efficiency may be a promising strategy for diabetes management, particularly when secondary bacterial infections are present. In this context, an antibiotic with beneficial immunomodulatory properties is of considerable interest.

Antibiotics are traditionally known for their antimicrobial properties, but their effects extend beyond antimicrobial activity. Previous studies have shown that antibiotics can also exert immunomodulatory effects, influencing the functions of phagocytic cells, including macrophages. For instance, tetracyclines such as oxytetracycline have been found to suppress the phagocytic capacity of macrophages [16,17]. On the other hand, certain fluoroquinolones [18,19,20] and cephalosporins [21] have enhanced macrophage phagocytosis and intracellular bacterial killing. The combination of sulfamethoxazole and trimethoprim has also been shown to enhance intracellular bactericidal activity in human monocyte-derived macrophages [22]. Additionally, ciprofloxacin has been demonstrated to suppress pro-inflammatory cytokine production, including TNF-α, IL-1, and IL-6 [23], while cefotaxime reduced IL-1 secretion but not TNF-α and IFN-γ [24]. Meanwhile, oxytetracycline and co-trimoxazole (sulfamethoxazole–trimethoprim) did not affect TNF-α, IL-1β, IL-6, and NOS expression of macrophages [25,26]. Although the immunomodulatory effects of antibiotics have been extensively researched, their effects on macrophage functions, particularly in the context of diabetes, remain an underexplored area.

The present study aimed to determine the effects of antibiotics on macrophage functions, including cytotoxicity, phagocytosis, bacterial intracellular killing, and pro-inflammatory mediator production under high glucose conditions. We focused on antibiotics that are commonly used in the treatment of diabetes-associated bacterial infections. The effects of four antibiotics, oxytetracycline, ciprofloxacin, sulfamethoxazole–trimethoprim, and cefotaxime, were assessed. Cefotaxime, sulfamethoxazole–trimethoprim, and ciprofloxacin are the first-line treatment of common bacterial infections in diabetic conditions [4,5]. Oxytetracycline has also been applied to treat diabetic foot infections [27]. Here, macrophages were cultured under prolonged high glucose exposure to mimic clinically relevant diabetes conditions. Thus, the present study highlights potential antibiotics with clinical benefits for the management of infections in diabetic patients.

## 2. Results

### 2.1. Minimum Inhibitory Concentration (MIC) and Cytotoxicity of Antibiotics on Murine Macrophage Cell Line, RAW 264.7, Under Low and High Glucose Levels

The minimum inhibitory concentration (MIC) values were determined to assess the antibacterial potency of each antibiotic against *Escherichia coli* ATCC 25922. It was observed that all the tested antibiotics (OTC, CIP, SXT, and CTX) demonstrated strong antibacterial activity, with CTX exhibiting the most antibacterial potency against Escherichia coli ATCC 25922 in this study, as shown in Table 1. The present results confirmed that the bacteria *Escherichia coli* ATCC 25922 was a sensitive bacteria according to Clinical and Laboratory Standards Institute guidelines [28]. These MIC values served as benchmarks for subsequent cytotoxicity assessments on macrophages treated with 1×, 2×, 4×, and 8× MIC concentrations under both low and high glucose conditions.

**Figure 1:**
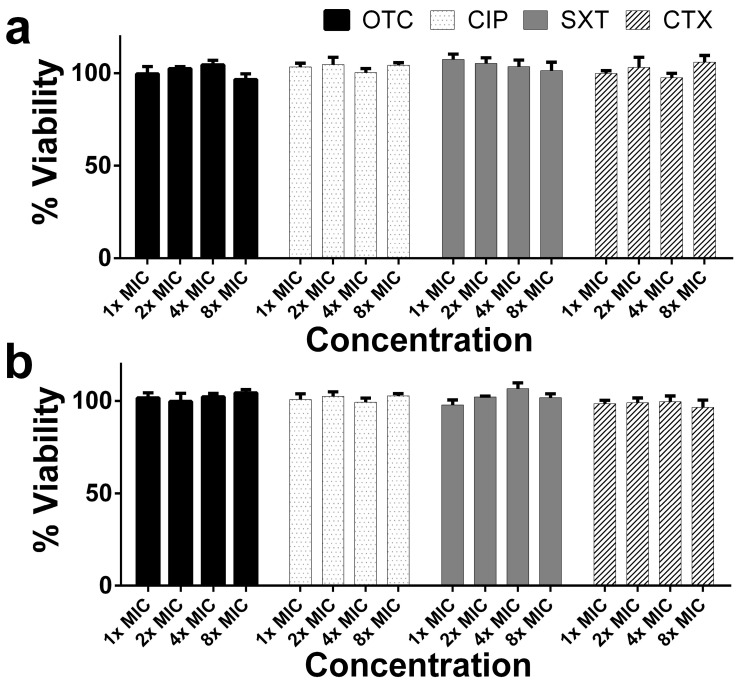
The cytotoxicity of antibiotics on murine macrophage cell lines, RAW 264.7 under low and high glucose levels. RAW 264.7 macrophages were incubated with antibiotics: oxytetracycline (OTC), ciprofloxacin (CIP), sulfamethoxazole/trimethoprim (SXT), and cefotaxime (CTX) at 1 time of MIC (1× MIC), 2 times of MIC (2× MIC), 4 times of MIC (4× MIC), and 8 times of MIC (8× MIC) under low glucose (**a**) and high glucose (**b**) conditions for 24 h. The percentage of cell viability was calculated by evaluating antibiotic cytotoxicity using the MTT assay. Each bar presents the mean ± SEM from three experiments.

The cytotoxicity was evaluated using the MTT assay on RAW 264.7 cells at concentrations ranging from 1× to 8× MIC under both low and high glucose conditions. Under low glucose, OTC treatments had no toxicity to the macrophage cells across all concentrations ranging from 1× to 8× MIC. All cell viabilities were not significantly different from the low glucose control group (*p* > 0.05). Cell viabilities of OTC were 99.64, 102.48, 104.46 and 96.53% at 1×, 2×, 4×, and 8× MIC, respectively. Treatments of CIP also exhibited no cytotoxic effects, with cell viabilities of 103.34, 104.69, 100.36, and 104.27% at the same concentration range. Similarly, SXT and CTX maintained cell viability at levels close to or slightly above 100%, showing no significant difference compared to the control group (*p* > 0.05). For SXT, cell viabilities were 107.36, 105.22, 103.55, and 101.39%, while CTX showed viabilities of 99.81, 103.03, 97.50, and 105.97% at 1×, 2×, 4×, and 8× MIC, respectively (Figure 1a). Under high glucose conditions, cell viability remained stable across all antibiotic concentrations, with only minor, statistically non-significant fluctuations. Cell viabilities of OTC under high glucose conditions were 101.62, 99.72, 102.25, and 104.32% at 1×, 2×, 4×, and 8× MIC, respectively, with no significant differences compared to the high glucose control group (*p* > 0.05). Cell viability in the CIP-treated group was comparable to the control group (*p* > 0.05), with values of 100.79%, 102.49%, 99.31%, and 102.78%. Varying MIC multiples of SXT and CTX revealed no toxicity to the macrophage cells under high glucose levels as compared to the control group (*p* > 0.05). Cell viabilities of SXT were 97.82, 102.18, 106.63, and 101.73%, and those for CTX were 98.59, 99.05, 99.64, and 96.60% at 1×, 2×, 4×, and 8× MIC, respectively (Figure 1b).

In addition, at both low and high glucose conditions, there were no significant differences in cell viability when compared to MIC multiples within each antibiotic (*p* > 0.05). It was revealed that antibiotics under low and high glucose levels generally maintained macrophage viability across all concentrations, indicating no cytotoxic effects under either glucose condition.

### 2.2. The Effect of Antibiotics on Macrophage Phagocytosis Under High Glucose Levels

**Figure 2:**
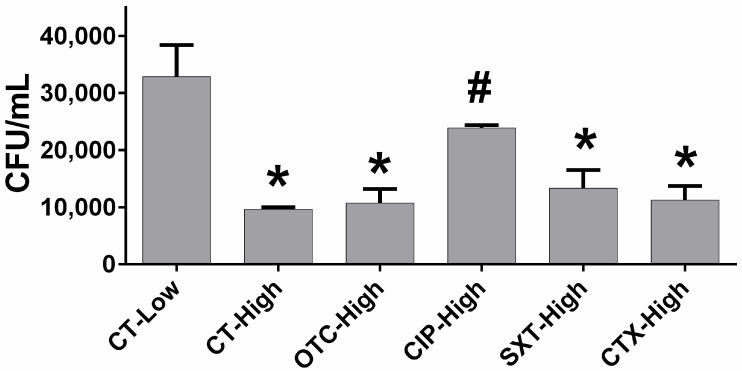
The effect of antibiotics on macrophage phagocytic activity under high glucose levels. RAW 264.7 macrophage cells were incubated for 24 h with designed conditions: (1) under low glucose without antibiotics (CT-Low), (2) under high glucose without antibiotics (CT-High), (3) under high glucose with OTC (OTC-high), (4) under high glucose with CIP (CIP-High), (5) under high glucose with SXT (SXT-high), (6) under high glucose with CTX (CTX-High). Then Escherichia coli ATCC 25922 were added and incubated for 60 min. The viable bacteria ingested by macrophages were cultured on a Mueller–Hinton agar plate, and the number of bacterial colonies was counted (CFU/mL). Each bar presents the mean ± SEM from three experiments. * *p* < 0.05 vs. CT-Low group, # *p* < 0.05 vs. CT-High group.

The effect of antibiotics on macrophage phagocytosis was analyzed by measuring the number of colony-forming units in the treated macrophages exposed to *Escherichia coli*. The present study revealed that the high glucose itself significantly reduced phagocytic activity, as evidenced by a decrease in CFUs from 32,866.67 ± 5567.86 CFU/mL in the low glucose control group to 9666.67 ± 333.33 CFU/mL in the high glucose control group (*p* < 0.05). Under high glucose, macrophages treated with OTC, SXT, and CTX exhibited reduced phagocytic activity compared to the low glucose control group (*p* < 0.05). However, their phagocytic activities were not significantly different from those observed in the high glucose control group (*p* > 0.05). The CFU counts of ingested bacteria for the OTC, SXT, and CTX groups were 10,750.00 ± 2428.13 CFU/mL, 13,316.67 ± 3193.79 CFU/mL, and 11,266.67 ± 2433.33 CFU/mL, respectively. Whereas the antibiotic treatment with CIP showed significantly higher CFU counts compared to the high glucose control group (*p* < 0.05). The CFU count of ingested bacteria in the CIP-treated group was comparable to the low glucose control group (*p* > 0.05), with a CFU count of 23,916.67 ± 463.98 CFU/mL. Also, the CFU counts for the CIP-treated group did not differ significantly from those treated with other antibiotics: OTC, SXT, and CTX (*p* > 0.05) (Figure 2).

### 2.3. The Effect of Antibiotics on Macrophage Bactericidal Activity Under High Glucose Levels

**Figure 3:**
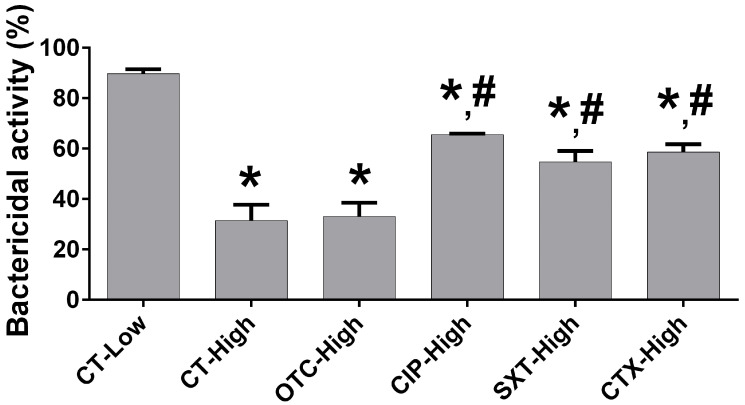
The effect of antibiotics on bactericidal activity of macrophages under high glucose levels. Macrophage RAW 264.7 cells were incubated for 24 h under designed experiments: (1) low glucose without antibiotics (CT-Low), (2) high glucose without antibiotics (CT-High), and high glucose with antibiotics, including (3) OTC (OTC-High), (4) CIP (CIP-High), (5) SXT (SXT-High), and (6) CTX (CTX-High). Afterward, Escherichia coli ATCC 25922 was added and incubated for 60 min and 24 h. The number of viable bacteria in macrophages at 60 min and 24 h were quantified (CFU/mL) by culturing on Mueller–Hinton agar. The percentage of bactericidal activity was calculated. Each bar presents the mean ± SEM from three experiments. * *p* < 0.05 vs. CT-Low group, # *p* < 0.05 vs. CT-High group.

Bactericidal activity was evaluated by assessing the capacity of macrophages to kill intracellular Escherichia coli by measuring the percentage reduction in bacterial load. Under high glucose, untreated macrophages exhibited a significantly reduced percentage of bactericidal activity, achieving only 31.48% bacterial killing compared to 89.73% in the low glucose control group (*p* < 0.05), highlighting the negative impact of hyperglycemia on bactericidal function. Furthermore, control macrophages cultured in low glucose exhibited the highest bactericidal activity among all groups of study (*p* < 0.05). Comparatively, the bactericidal activity of macrophages in the low glucose control group was significantly higher, approximately 2.85-fold, than in the high glucose control group. It also increased by about 2.71, 1.64, 1.53, and 1.37-fold compared to macrophages incubated with OTC, CIP, SXT, and CTX, respectively. Importantly, under high glucose, treatments with CIP, SXT, and CTX significantly improved the bactericidal activity, achieving 65.51 ± 0.46, 54.70 ± 4.37%, and 58.65 ± 3.05%, respectively, which were markedly better than the high glucose control group (*p* < 0.05). OTC treatment, however, showed minimal improvement, with bactericidal activity of 33.07 ± 5.43%, comparable to the high glucose control group (*p* > 0.05) (Figure 3).

### 2.4. The Effect of Antibiotics on mRNA Expression of Pro-Inflammatory Mediators Under High Glucose Levels

**Figure 4:**
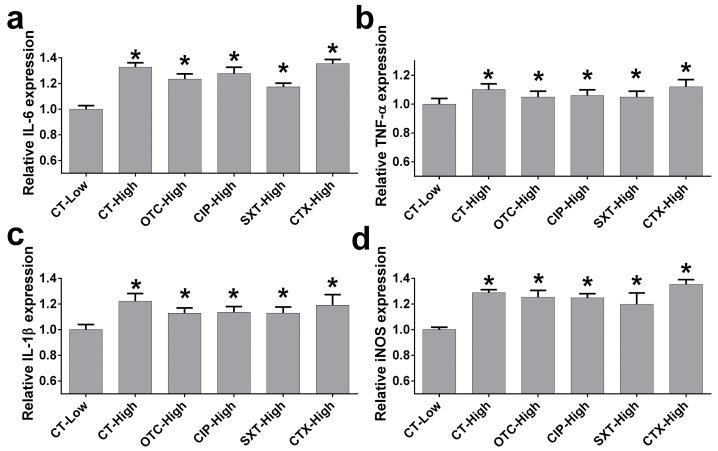
The effect of antibiotics on pro-inflammatory mediator expressions under high glucose levels. The mRNA expressions of pro-inflammatory mediators, IL-6 (**a**), TNF-α (**b**), IL-1β (**c**), and iNOS (**d**), were analyzed using the RT-PCR method. Samples were collected from RAW 264.7 macrophages were exposed to designed conditions for 24 h: (1) low glucose without antibiotics (CT-Low); (2) high glucose without antibiotics (CT-High); and high glucose with (3) OTC (OTC-High), (4) CIP (CIP-High), (5) SXT (SXT-High), and (6) CTX (CTX-High). Each bar presents the mean ± SEM from three experiments. * *p* < 0.05 vs. CT-Low group.

To evaluate the effect of antibiotics on the production of inflammatory-associated mediators, the RT-PCR assay was performed. The mRNA expressions of pro-inflammatory mediator markers, IL-6, TNF-α, IL-1β, and iNOS were determined in the murine macrophage cell lines RAW 264.7 after designed treatments. The results showed that the expression of IL-6 mRNA in the high glucose control was significantly increased compared to the low glucose control group (*p* < 0.05). Similarly, under high glucose, the macrophages treated with OTC, CIP, SXT, and CTX exhibited higher levels of IL-6 expression compared to the low glucose control (*p* < 0.05). Among groups under high glucose conditions, the high glucose control or treatments of OTC, CIP, SXT, and CTX, no significant differences in IL-6 mRNA expression were observed (*p* > 0.05). For TNF-α mRNA, a significant increase was observed in the high glucose control group compared to the low glucose control group (*p* < 0.05). Also, macrophages treated with the four antibiotics under high glucose conditions exhibited significantly higher TNF-α levels than the low glucose control group (*p* < 0.05). No significant differences were observed in TNF-α expression among those groups of macrophages under high glucose conditions (*p* > 0.05). In the IL-1β and iNOS mRNA expressions, macrophages under high glucose without antibiotics showed significantly higher expression levels compared to the low glucose control group (*p* < 0.05). Accordingly, treatment with OTC, CIP, SXT, or CTX under high glucose conditions resulted in significantly elevated IL-1β and iNOS expressions compared to the low glucose control group (*p* < 0.05). There were no significant differences in these markers between the high glucose control and antibiotic-treated groups or among antibiotics treatment groups (*p* > 0.05) (Figure 4).

## 3. Discussion

The present study demonstrated how high glucose conditions and antibiotic treatments impacted macrophage functions, including cytotoxicity, phagocytic activity, bactericidal efficiency, and pro-inflammatory mediator expression. Antibiotics (OTC, CIP, SXT, CTX) at 1× to 8× MIC concentrations showed no significant cytotoxic effects on RAW 264.7 macrophages under both low and high glucose conditions. High glucose significantly impaired macrophage phagocytosis, as indicated by a marked reduction in CFU of ingested bacteria compared to low glucose conditions. CIP was the only antibiotic that significantly improved phagocytic activity under high glucose. High glucose severely reduced macrophage bactericidal activity, while antibiotic treatments partially improved bactericidal function. Moreover, high glucose increased the pro-inflammatory cytokine expressions of *IL-6*, *TNF-α*, *IL-1β*, and *iNOS* compared to low glucose control. Antibiotics did not significantly alter these elevated levels.

Although all four antibiotics are commonly recommended for treating bacterial infection in diabetic patients [4,5], their cytotoxic effects on immune cells have not been well studied. These drugs are generally considered safe, which aligns with the present study’s findings that all antibiotic concentrations ranging from 1× to 8× MIC did not show toxicity to macrophages under both low and high glucose conditions. Previous studies have demonstrated that OTC [17], CTX [29], SXT [30], and CIP [31] induced cytotoxicity in epithelial cells and macrophages. These differences suggest that, in previous studies, the drugs induced cytotoxicity only at much higher concentrations compared to those used in the present study.

Based on non-toxic concentration and to avoid underexposure to antibiotics, the concentration at two times MIC of each antibiotic was used in the present study of macrophage phagocytosis, bactericidal activity, and pro-inflammatory mediator production. In the present results, hyperglycemia significantly impaired macrophage phagocytic activity, consistent with previous findings reporting a reduction in phagocytosis under hyperglycemic conditions [11]. The impaired uptake of opsonized particles and decreased expression of key phagocytic receptors in hyperglycemia were also reported [15], highlighting the consistent negative impact of high glucose levels on macrophage function. However, CIP significantly enhanced phagocytic activity in this study, aligning with previous reports of fluoroquinolones improving macrophage phagocytic function [18,20]. In contrast, treatment with OTC showed no enhancement in macrophage phagocytosis, consistent with previous findings demonstrating its suppressive effect on phagocytic activity [16,17]. Additionally, in the present study, treatments with SXT and CTX had no impact on macrophage phagocytosis under hyperglycemic conditions, which aligns with the previous demonstration indicating no change in phagocytosis with these treatments [32,33]. The present findings imply that the antibiotic-specific modulation of phagocytic function may depend on drug characteristics such as cellular uptake and immunomodulatory properties. The enhanced effect of CIP observed in this study suggests that CIP exhibits relatively higher cellular penetration compared to other antibiotics [34,35]. Additionally, CIP may activate the PI3K/Akt pathway [36], which serves as an important mediator in the process of phagocytosis [37]. Additionally, OTC [38], SMX [39], and CTX [40] presented inhibition of Akt signaling. It was noted that the high glucose concentration exhibited suppression of PI3K/Akt signaling [41], which aligned with impaired phagocytosis in the macrophages of the present study. Moreover, the hyperglycemia-impaired PI3K/Akt signaling has been linked to insulin resistance and Type II diabetes [42].

Hyperglycemia also suppressed macrophage bactericidal activity in the present study, as evidenced by decreased intracellular bacterial killing. This finding was consistent with previous reports that high glucose could induce impaired bactericidal capacity in macrophages, leading to an increased risk of developing infections [10,11]. In this study, CIP, SXT, and CTX presented an enhanced effect on macrophage bactericidal activity under high glucose conditions. These results were consistent with previous findings showing that CIP improved intracellular bacterial killing in macrophages [19,22] and that SXT and CTX increased macrophage-mediated killing of intracellular *Salmonella* species [21,22]. However, the present study observed that OTC did not improve bactericidal activity, aligning with its limited phagocytic enhancement. The present study suggested that CIP, SXT, and CTX effectively alleviated hyperglycemia-induced suppression of bactericidal capacity, whereas OTC did not. These differences may result from variations in drug ability to phagocyte accumulation, indicating that the intracellular accumulation of OTC was relatively lower than that of CIP, SXT, and CTX [34,35]. Moreover, differences in mechanisms of action, such as bactericidal antibiotics (CIP, SXT, and CTX) versus bacteriostatic antibiotics (OTC), may explain the minimal improvement in bactericidal activity observed with OTC in the present study. Furthermore, in this study, it was observed that SXT and CTX effectively improved bactericidal activity under high glucose conditions but not for phagocytic activity. This suggests that their bactericidal mechanisms did not depend on enhanced phagocytosis but instead on intracellular bacterial clearance mechanisms [43].

The present findings showed that hyperglycemia promoted the pro-inflammatory phenotype in macrophages, which was characterized by increased expressions of marker pro-inflammatory mediators, including TNF-α, IL-6, IL-1β, and iNOS. This was in agreement with previous reports that the high glucose environment demonstrated induction of the pro-inflammatory state in macrophages [10,11,12,13]. This may further promote sustained polarization of M1 macrophages, leading to prolonged inflammation [14]. This sustained inflammation has been linked to impaired phagocytosis and bactericidal capacity, exacerbating susceptibility to infection in diabetic patients [10,44,45]. The present results exhibited that the four antibiotics under high glucose did not affect the pro-inflammatory cytokine expression. This was in agreement with previous studies, which found that OTC [26] and SXT [25] treatments had no impact on pro-inflammatory production. In contrast, treatments with CIP and CTX showed the suppression of the pro-inflammatory cytokine expressions, including IL-6, IL-1β, and TNF-α [23,24]. These differences may arise from variations in experimental conditions, such as cytokine measurement timelines or macrophage sources. In the present study, the lack of significant cytokine modulation by antibiotics suggested their effects were primarily antibacterial rather than anti-inflammatory in this context.

In this in vitro study, under high glucose conditions, RAW 264.7 murine macrophage cell lines were cultured with 25 mmol/L glucose for a prolonged period, simulating glucose levels typical in diabetic patients (normal range: 5.6–6.9 mmol/L, WHO). The present results demonstrated that the macrophages exposed to high glucose exhibited phenotypes and functions similar to those observed in the diabetic patient, including pro-inflammatory characteristics and impaired capacities of phagocytosis and cellular bacterial killing [10,44,45]. This suggests that this model is well-suited for investigating immune dysfunctions linked to diabetes. However, studies conducted within an immune in vivo model are necessary to evaluate the efficacy and toxicity of antibiotics for potential use in diabetic treatment. The cytokine protein and activity measurements were not addressed in the present study, restricting the direct applicability of findings to clinical aspects. Although, in the present results, CIP may be a promising option to enhance macrophage phagocytosis and bactericidal activity, the underlying molecular mechanism of the enhanced phagocytic and bactericidal effects should be elucidated. Addressing these gaps in future research will be critical for optimizing antibiotic use in diabetic immune dysfunction and improving infection outcomes.

## 4. Materials and Methods

### 4.1. Bacterial Culture

*Escherichia coli* ATCC 25922 was obtained from the Department of Medical Sciences, Ministry of Public Health, Thailand. The strain was preserved in Tryptic Soy Broth (Becton Dickinson and Company, Sparks, MD, USA) with 10% glycerol and stored at –80 °C until use. The bacteria were cultured on Mueller–Hinton agar (Himedia Laboratories, Dindhori, Nashik, India), and a single colony was selected to prepare *Escherichia coli* concentration at No. 0.5 McFarland (1 × 10^8^ CFU/mL) for the subsequent experiments.

### 4.2. Macrophage Culture

**Figure 5:**
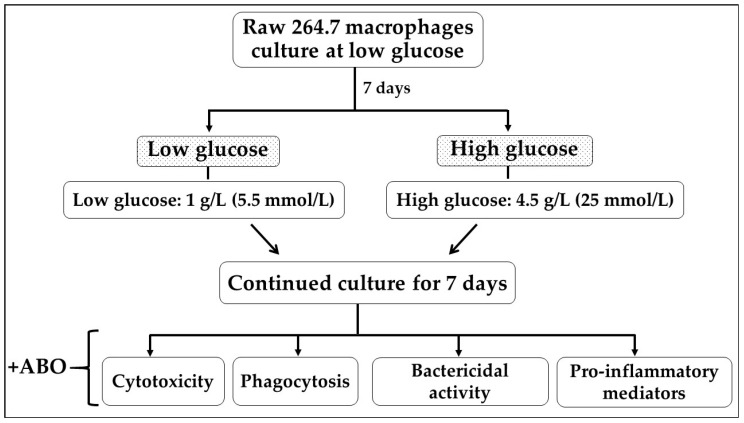
Schematic illustration of experimental setup. RAW 264.7 macrophages were cultured for 7 days in a low-glucose medium and then divided into low (5.5 mmol/L) and high (25 mmol/L) glucose conditions for another 7 days. Antibiotics were added, and their impact on cytotoxicity, phagocytosis, bactericidal activity, and pro-inflammatory mediator production was evaluated.

The murine macrophage cell lines RAW 264.7 were obtained from ACTT (Manassas, VA, USA). All experiments began with frozen cell stocks, and the cells were evaluated based on their morphology and growth kinetics to confirm their characteristics. At the beginning, RAW 264.7 macrophages were cultured in Dulbecco’s Modified Eagle Medium (DMEM) with 1 g/L glucose (Low glucose) (Gibco BRL Life Technologies, Grand Island, NY, USA). The medium was supplemented with 10 mM N-2-hydroxyethylpiperazine-N-2-ethanesulfonic acid (HEPES) pH 7.35 (AppliChem GmbH, Darmstadt, Germany) and 10% fetal calf serum (Global Life Sciences Solutions Austria GmbH & Co KG, Pasching, Austria) without antibiotics. The RAW 264.7 macrophages were cultured under low glucose medium at an atmosphere of 5% CO_2_, 37 °C for 7 days. Then, the cultured cells were divided into two conditions: low and high glucose. Under low glucose, the macrophage cells were cultured DMEM containing 1 g/L glucose (5.5 mmol/L glucose). Meanwhile, under high glucose conditions, the macrophage cells were cultured DMEM containing 4.5 g/L glucose (25 mmol/L glucose) (Gibco BRL Life Technologies, Grand Island, NY, USA). Next, cultured cells in both conditions were maintained at 37 °C in a 5% CO_2_ atmosphere for 7 days without antibiotics before proceeding to the subsequent experiments. A detailed schematic of the experimental setup was shown in Figure 5.

### 4.3. Minimum Inhibitory Concentration Determination

A broth microdilution method was used to determine the minimum inhibitory concentration (MIC) for four antibiotics: OTC (Sigma-Aldrich Pte. Ltd., Singapore), CIP (Sigma-Aldrich Pte. Ltd., Singapore), SXT (Sigma-Aldrich Pte. Ltd., Singapore), and CTX (Sigma-Aldrich Pte. Ltd., Singapore) according to Clinical and Laboratory Standards Institute guidelines (CSLI 2013) [46]. In brief, serial two-fold dilutions of all antibiotics with the following concentrations (μg/mL): OTC (0.0156-8), CIP (0.0156-8), SXT (0.2968/0.0156-152/8), and CTX (0.0156-8) were prepared with Mueller–Hinton II broth (MHB II) (Becton Dickinson and Company, Sparks, MD, USA). Then, 200 μL of each antibiotic dilution was added into 96-well microplates. A 10 μL of *Escherichia coli* ATCC 25922 solution at a concentration of 10^7^ CFU/mL was added, achieving a final concentration of 10^5^ CFU/mL of bacteria in each well. The plates were incubated for 16–20 h at 35 °C. The MIC was recorded as the lowest concentration that visibly inhibited bacterial growth in the well, as observed with the unaided eye.

### 4.4. MTT Assay

An assay was performed to investigate the cytotoxicity of antibiotics on macrophage cells. The murine macrophage cell lines RAW 264.7 were seeded at a density of 1 × 10^4^ cells/well into a 96-well plate with low and high glucose medium and allowed to adhere overnight. The old medium was then replaced with a new complete medium containing the various concentrations of four antibiotics (OTC, CIP, SXT, and CTX) ranging from 1× to 8× MIC. The old medium was discarded and then incubated with the medium containing 12 mM MTT dye (Sigma Chemical, St. Louis, MO, USA) at 37 °C for 3 h. A formazan crystal was dissolved in 100% DMSO (Loba Chemie, Colaba, Mumbai, India) for absorbance reading with a microplate reader at a wavelength of 570 nm.

### 4.5. Macrophage Phagocytosis and Bacterial Killing Assay

An assay was performed to determine the activities of phagocytic and bacterial killing in macrophages by modifying the method, as described previously [47]. RAW 264.7 murine macrophage cells at a density of 1 × 10^6^ cells/well were cultured onto a six-well plate overnight. The macrophage cells were then incubated with designed conditions: (1) low glucose without antibiotics (the low glucose control); (2) high glucose without antibiotics (the high glucose control); (3) high glucose with 2× MIC of OTC, high glucose with 2× MIC of CIP, high glucose with 2× MIC of SXT, and high glucose with 2× MIC of CTX for 24 h. After that, the treated macrophages were trypsinized with 0.25% trysin- EDTA (Gibco BRL Life Technologies, Grand Island, NY, USA) and reseeded into a 24-well plate at a density of 2 × 10^5^ cells/well and allowed to adhere overnight. *Escherichia coli* ATCC 25922 was added into the well at multiplicity of infection (MOI) 1:10 (2 × 10^6^ CFU/mL). The co-culture of treated macrophages/*Escherichia coli* was incubated at 37 °C, 5% CO_2_ for 60 min and 24 h. The sample was next washed with phosphate-buffered saline (PBS) pH 7.4 5 times to discard extracellular bacteria (the bacteria count was not found in the fifth washed PBS) and lysed with 0.1% Triton-X (Loba Chemie, Colaba, Mumbai, India). The sample was 10-fold serial dilution using 0.85% NaCl and spread onto Mueller–Hinton agar. The bacteria counts as the number of colony-forming units per milliliter (CFU/mL) was measured after the plate was incubated at 37 °C for 20 h. The phagocytic activity was expressed as the number of viable bacteria ingested by macrophages at 60 min of incubation (CFU/mL). The percentage of bactericidal activity was calculated as follows:

$$\begin{array}{l} Percentage\;of\;bactericidal\;activity\\ \;=100\;-100\times [\;The\;number\;of\;remaining\;viable\;bacteria\;in\;macrophages\;at\;24\;h\;(CFU/mL)\\ \;÷The\;number\;of\;viable\;bacteria\;ingested\;by\;macrophages\;at\;60\;min\;(CFU/mL)] \end{array}$$

### 4.6. Quantitative RT-PCR Analysis

To determine the effects of antibiotics on mRNA expression of pro-inflammatory mediator markers, including *IL-6*, *TNF-α*, *IL-1β*, and *iNOS*, an assay was performed as previously described [48]. The murine macrophage cells, RAW 264.7, were seeded at a density of 1 × 10^6^ cells/well and allowed to adhere overnight. The macrophage cells were incubated under the following conditions for 24 h: (1) low glucose without antibiotics, (2) high glucose without antibiotics, (3) high glucose with OTC at 2× MIC, (4) high glucose with CIP at 2× MIC, (5) high glucose with SXT at 2× MIC, and (6) high glucose with CTX at 2× MIC. After that, the treated cells were subjected to RT-PCR analysis. Total RNA was extracted using the Nucleospin RNA kit (Macherey-Nagel, Düren, Germany) and reverse-transcribed into cDNA with the ReverTra Ace qPCR RT Kit (TOYOBO, Tokyo, Japan). Gene expression was quantified using the QuantStudio™3 Real-Time PCR system (Applied Biosystems, Waltham, MA, USA) with Maxima Sybr Green qPCR Mastermix (Thermo Inc, Waltham, MA, USA). Expression levels were normalized to GAPDH as the reference gene. Primer sequences used for amplification are listed in Table 2.

### 4.7. Statistical Analysis

Data were presented as mean ± SEM of three independent experiments. The means of multiple groups were compared by one-way ANOVA with Tukey’s multiple comparisons post hoc test. The results were considered to be significant at *p* < 0.05.

## 5. Conclusions

In the present study, no cytotoxicity was observed with all antibiotics, even at the highest concentration test under both high glucose and low glucose levels. The present study demonstrated the deleterious effects of hyperglycemia on macrophage functions, including reduced phagocytosis, impaired bactericidal activity, and induced pro-inflammatory cytokine production. Importantly, CIP was shown to significantly improve macrophage phagocytosis and bactericidal activity under high glucose conditions, while SXT and CTX moderately enhanced bactericidal activity without affecting pro-inflammatory cytokine expression. This study provided foundational evidence for the immunomodulatory properties of antibiotics under high glucose, potentially improving infection management strategies for diabetic patients. The findings emphasized the potential of CIP as a therapeutic option for current diabetic infection management strategies.

## Figures and Tables

**Table 1:** Minimum inhibitory concentration (MIC) of antibiotics against Escherichia coli ATCC 25922 using broth microdilution method.

Antibiotics	Results of Minimum Inhibitory Concentration (MIC, μg/mL) *
	*Escherichia coli* ATCC 25922
Oxytetracycline (OTC)	1
Ciprofloxacin (CIP)	0.125
Sulfamethoxazole/trimethoprim (SXT) ^#^	0.125/2.375
Cefotaxime (CTX)	0.03125

* MIC—minimum inhibitory concentration; ^#^ sulfamethoxazole/trimethoprim ratio followed the CLSI recommendation.

**Table 2:** Primers used for RT-PCR.

Gene	Forward	Reverse	References
*IL-6*	TCCATCCAGTTGCCTTCTTG	CATTTCCACGATTTCCCAGAG	[49]
*TNF-α*	TTGAGTGCCAATTCGATGATG	GAGGGCTTGTTGAGATGATGC	[49]
*IL-1β*	GACGGACCCCAAAAGATGAAG	CTCCACAGCCACAATGAGTGA	[49]
*iNOS*	TTTGTGCGAAGTGTCAGTGG	CCCTTTGTGCTGGGAGTCA	[50]
*GAPDH*	GGCATTGTGGAAGGGCTCAT	GACACATTGGGGGTAGGAACAC	[49]

## Data Availability

All data generated or analyzed during this study are included in this published article.
